# Systemic PD149163, a neurotensin receptor 1 agonist, decreases methamphetamine self-administration in DBA/2J mice without causing excessive sedation

**DOI:** 10.1371/journal.pone.0180710

**Published:** 2017-07-07

**Authors:** Amanda L. Sharpe, Erika Varela, Michael J. Beckstead

**Affiliations:** 1 Department of Pharmaceutical Sciences, Feik School of Pharmacy, University of the Incarnate Word, San Antonio, Texas, United States of America; 2 Department of Cellular and Integrative Physiology, UT Health, San Antonio, Texas, United States of America; 3 Center for Biomedical Neuroscience, UT Health, San Antonio, Texas, United States of America; Washington State University College of Veterinary Medicine, UNITED STATES

## Abstract

Methamphetamine (METH) is a psychostimulant that exhibits significant abuse potential. Although METH addiction is a major health and societal concern, no drug is currently approved for its therapeutic management. METH activates the central dopaminergic “reward” circuitry, and with repeated use increases levels of the neuromodulatory peptide neurotensin in the nucleus accumbens and ventral tegmental area. Previous studies in rats suggest that neurotensin agonism decreases METH self-administration, but these studies did not examine the effect of neurotensin agonism on the pattern of self-administration or open field locomotion. In our studies, we established intravenous METH self-administration in male, DBA/2J mice (fixed ratio 3, 2 hr sessions) and examined the effect of pretreatment with the NTS1 receptor agonist PD149163 on METH self-administration behavior. Locomotion following PD149163 was also measured up to 2 hours after injection on a rotarod and in an open field. Pretreatment with PD149163 (0.05 and 0.10 mg/kg, s.c.) significantly decreased METH self-administration. The pattern of responding suggested that PD149163 decreased motivation to self-administer METH initially in the session with more normal intake in the second hour of access. Voluntary movement in the open-field was significantly decreased by both 0.05 and 0.10 mg/kg (s.c.) PD149163 from 10–120 minutes after injection, but rotarod performance suggested that PD149163 did not cause frank sedation. These results suggest that a systemically delivered NTS1 receptor agonist decreases METH self-administration in mice. The pattern of self-administration suggests that PD149163 may acutely decrease motivation to self-administer METH before the drug is experienced, but cannot rule out that depression of voluntary movement plays a role in the decreased self-administration.

## Introduction

Methamphetamine (METH) is a psychomotor stimulant with therapeutic utility, but its liability as a drug of abuse is a major societal problem. There are currently no drugs approved by the U.S. FDA for treatment of METH addiction, suggesting that a better understanding of receptor systems capable of modulating drug intake is necessary. Acutely, METH competes with dopamine for uptake at plasmalemmal and vesicular transporters [1], elevating extracellular levels of the neurotransmitter and producing behavioral reinforcement [2]. Chronic METH use produces adaptations to many neurotransmitter systems including those involving the peptide neurotensin, a tridecapeptide and neuromodulator first identified in the bovine hypothalamus over 40 years ago [3]. Neurotensin input to the dopaminergic system is strong and originates predominantly in brain regions that have been implicated in natural and drug reward processes [4–5]. METH administration is correlated with an increase in extracellular neurotensin, with larger effects observed following self-administration versus experimenter-delivered controls [6–7]. Behaviorally, neurotensin signaling modulates dopamine-related processes such as cognition, food intake, and the effects of psychostimulants [8–11]. Dopaminergic neurons and their downstream targets are known to express surface neurotensin NTS1 receptors [12–14], and rats trained to discriminate a neurotensin receptor agonist show full generalization to the D2-like receptor antagonist haloperidol [15]. Neurotensin receptor agonism is known to decrease self-administration of food [16–19], nicotine [20] and METH [7].

Central neurotensin levels are responsive to METH self-administration, with levels increasing during METH use and decreasing during extinction [21]. Additionally, neurotensin agonism both during current METH self-administration [7] and during extinction [21] decreases responding for METH. However, one caveat to these previous studies is that the subjects in each case were food-deprived. Since neurotensin agonists also affect food intake [16–19] and differentially elicit release of norepinephrine in fasted state [22] it is possible that the food-restriction employed in these studies biased the findings to include effects on both feeding and METH self-administration.

Thus, the purpose of these studies was to investigate the effect of a NTS1 receptor agonist (PD149163) on operant METH self-administration in a non-food deprived mouse model. We examined patterns of METH self-administration and locomotor activity to investigate how PD149163 affects the initial motivation to self-administer METH, as well as operant responding as the mice consumed the drug.

## Materials and methods

### Animals

Male DBA/2J mice (6–10 weeks on arrival; Jackson Labs, Bar Harbor, ME) were housed in polycarbonate home cages with rodent bedding. The vivarium was on a reverse light cycle (14:10) with lights off from 09:00–19:00. Food and water were available ad libitum except as stated during food training. Mice were group housed until surgery, and were individually housed with a cotton nestlet (Shred-A-Bed) after surgery. All protocols were approved by the UTHSCSA Animal Care and Use Committee (Protocol 12066x), and mice were cared for consistent with *The Guide for the Care and Use of Laboratory Animals*. Humane endpoints (loss of >20% body weight, infection, etc) were in place for guiding decisions on the need for euthanasia throughout the course of the experiments. For surgery, mice were anesthetized with isoflurane and were pro-actively treated for possible pain and infection post-surgery with carprofen and ticarcillin.

### Open field locomotion

Mice (n = 6) naïve to METH were used to assess locomotor effects of PD149163 (0, 0.05, and 0.1 mg/kg) injected subcutaneously. All locomotor sessions were conducted at approximately 11:00 am (2 hours after lights off in the vivarium) under red light and were 30 minutes in length. Mice were transported to the behavioral suite at least 30 minutes before testing. Data were collected on a personal computer running Opto-Varimex AutoTracker software (Columbus Instruments, Columbus, OH). Mice were habituated to the open field locomotor chambers (16” x 16”, Columbus Instruments) for one day before testing began. For each of the two weeks of testing, mice received no injection on day 1, saline on days 2 and 4 and either the low or high dose of PD149163 (0.05 or 0.1 mg/kg, order counterbalanced across mice) before the session on days 3 and 5. In week 1 injections were given immediately before each mouse was placed in the locomotor chamber, and in week 2 the injections were given 90 minutes before placement in the locomotor chamber. Following the 30-minute locomotor session each day, the mice were returned to the vivarium.

### Rotarod

To test the effect of PD149163 (0, 0.05, 0.1 mg/kg) on directed locomotion, performance on a fixed speed rotarod was measured. Using methodology very similar to previously published reports [11], mice (DBA/2J adult males, n = 6) were trained in up to 8 trials to stay on the fixed speed rotarod (20 rpm, Med Associates, Fairfax, VT). Training was ended when the mouse was able to stay on the rotarod for a minimum of 180 s, and one mouse who did not meet criteria by the 8^th^ trial was dropped from the study. For the test days, each mouse received an injection (0, 0.05, or 0.1 mg/kg PD149163) and performance on the fixed speed rotarod was tested at 0, 30, 60, 90, and 120 minutes after injection. Each mouse received each dose, and testing was run every other day.

### Surgery

For surgical placement of a venous catheter, mice were anesthetized with isoflurane (1.5–3%, 1.5 L/min O_2_). All methods were similar to that previously published [23]. Venous catheters were constructed in house from micro-renathane tubing (0.025” o.d., 0.012” i.d.) with a silicone bead located approximately 10 mm from the end of the tubing. The catheter was inserted into the right jugular vein through a ventral incision, and surgical silk sutures were used to anchor the catheter to the vein above and below the silicone bead. Blood was aspirated through the catheter after placement and suturing to assure patency and proper placement in the vein. The micro-renathane tubing was then tunneled subcutaneously to the dorsal side where it connected to a length of 27-gauge hypodermic tubing that was bent at a 90° angle. The hypodermic tubing was threaded through the shaft of a nylon screw that exited the dorsal incision site between the scapulae. A polyester felt mesh (Surgical mesh, Brookfield, CT) in the shape of an oval at the base of the nylon screw was positioned subcutaneously so that the tissue would anchor the catheter to the exit site. Dissolvable sutures were used to close the ventral and dorsal incision sites. The hypodermic tubing external to the animal was occluded by a short piece of heat-fused PE20 tubing to decrease incidence of clotting and infection. All mice received carprofen and ticarcillin injections post-surgery to prevent infection and to alleviate pain. Catheters were flushed daily with approximately 0.025 ml of heparinized saline (30 units/ml) beginning 3 days after surgery.

### Operant self-administration

Before surgery to place the venous catheter, mice (n = 15) were trained to nose poke in operant self-administration chambers (Lafayette Instruments, Lafayette, IN) for delivery of a sucrose pellet. The operant chambers had 2 nose poke holes located on one side of the modular chamber, with stimulus lights contained within the holes that were used to designate the active side (counterbalanced across chambers). A receptacle was located between the nose pokes and sucrose pellets (20 mg, BioServe precision pellets) were delivered there during food training. A light within the receptacle was used to signify delivery of a reinforcer. Each chamber was located in a sound-attenuating cabinet with a fan to mask external noises. Mice were initially trained (two sessions of 45 min each) to approach the receptacle following delivery of a sucrose pellet that was delivered on a variable interval (average 45 s, range 15–74 s), followed by one session (60 minutes) at a fixed ratio 1 (FR1) in the correct side nose poke for delivery of a sucrose pellet. After these initial three training sessions, mice underwent surgery to place an intravenous catheter for METH self-administration (see section 2.3 for details).

After allowing at least 7 days to recover from surgery, mice (n = 11) were initiated on daily operant sessions to train self-administration of METH. Four mice were lost during or after surgery due to inability to place venous catheter or inability to recover from anesthesia immediately following the surgery. Over 6–10 sessions, mice were manually increased from an FR1 to an FR3 for METH based on performance (number of reinforcers and correct-side responding). Completion of the FR resulted in extinguishing the stimulus light in the nose poke, infusion of METH at a dose of 0.05 mg/kg/infusion (assuming a 28 g mouse, infused over 2 s in a volume of 12 μl), and began a 15 s time out during which responding was not reinforced. Throughout the session responding in the non-active hole was not reinforced, but the responding was recorded. At the end of the training sessions, mice (n = 8, 3 mice did not successfully acquire METH self-administration) were all performing on an FR3 for each infusion of METH, with 70% of total responses in the METH-associated nose poke hole.

### Drugs

METH hydrochloride was provided as a generous gift from the National Institute on Drug Abuse drug supply program (Bethesda, MD). PD149163 was purchased from Sigma-Aldrich (St. Louis, MO). All drug solutions for injection used sterile saline as the diluent.

### Statistics

GraphPad Prism (La Jolla, CA) was used to conduct all statistical analyses. Data are presented as mean ± SEM unless otherwise stated. A paired t-test was used to compare saline days immediately preceding PD149163 injections to determine if there was any statistically significant difference between intake. One-way repeated measures analyses of variance (ANOVAs) were used to determine if self-administration behaviors were significantly different following pre-treatment with saline (average of 2 saline days) and PD149163 (0.05 and 0.1 mg/kg). Two-way repeated measures ANOVAs were used to determine main effects of drug treatment and time on locomotor activity. Dunnett’s posthoc test was used when applicable. For all analyses, α was set at 0.05 a priori.

## Results

### Self-administration

**Fig 1:**
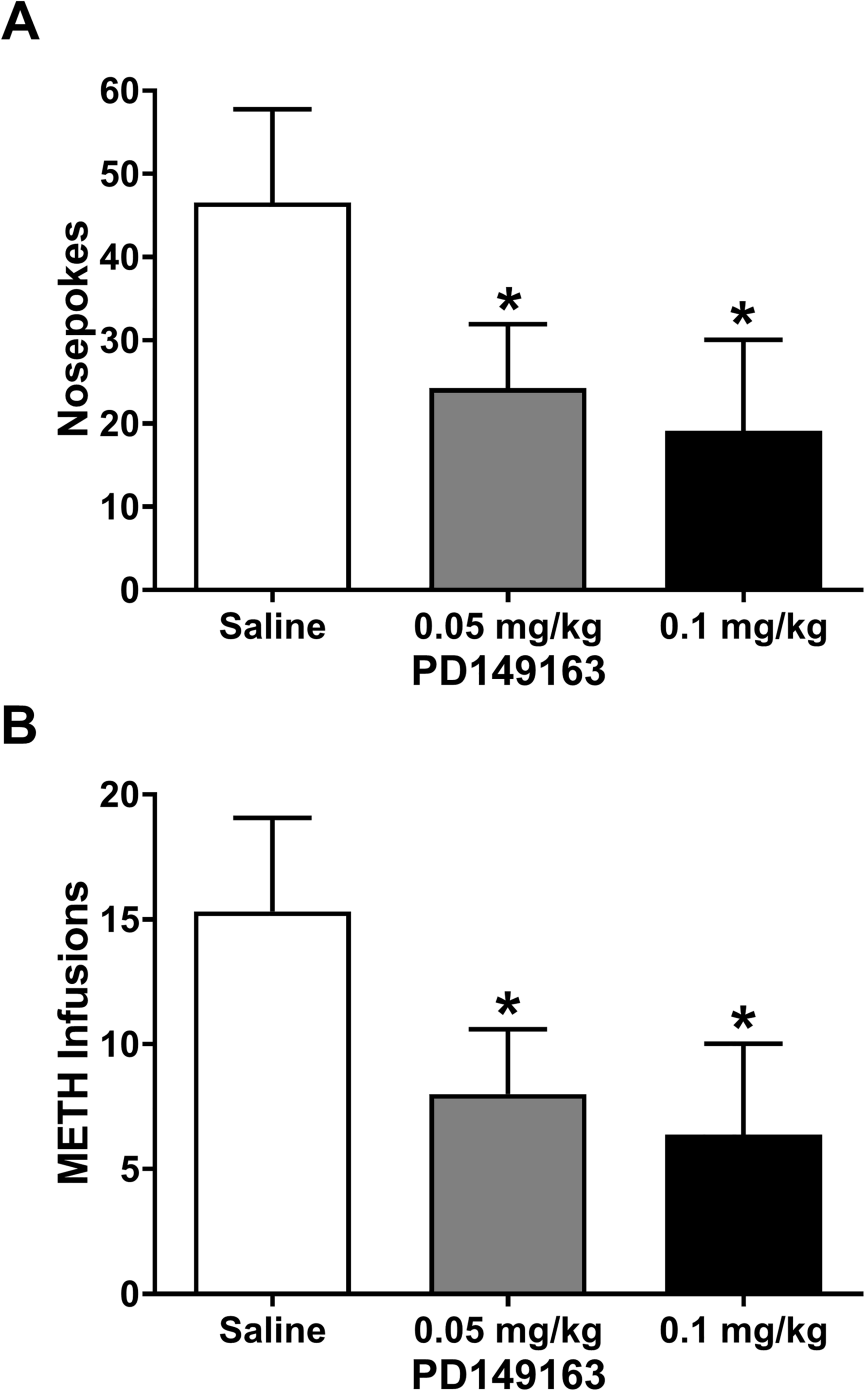
PD149163 decreases operant responding for METH in mice. (A) Systemic injection (s.c.) of both doses of PD149163 resulted in a significant decrease in nose pokes (FR3) made during 2-hour operant METH self-administration sessions. * *P* < 0.05 versus saline, Dunnett’s multiple comparison test. (B) Both doses of PD149163 also produced a significant decrease in number of METH infusions. * *P* < 0.05 versus saline, Dunnett’s multiple comparison test.

Mice acquired self-administration of METH with an average intake of 0.78 ± 0.22 mg/kg (14.6 ± 4.3 infusions) over the two days before their first injection. Mice were injected (s.c.) with 0, 0.05, or 0.1 mg/kg PD149163, with a saline injection on the days immediately preceding the PD149163 injections. A paired t-test comparing the number of active nose poke responses for the two saline injection days showed no significant difference in responding (*P* = 0.71), so the data from these two days were averaged for further analysis. There was a significant main effect of PD149163 on the number of active nose poke responses (Fig 1A; F_2,14_ = 5.8, *P* = 0.014) and the number of METH infusions earned (Fig 1B; F_2,14_ = 5.6, *P* = 0.017). Dunnett’s posthoc analysis revealed a significant decrease in number of active nose pokes and infusions earned at both doses of PD149163 tested when compared to saline (Fig 1A and 1B). There was no significant main effect of PD149163 on the number of incorrect side nose pokes (*P* = 0.284) or receptacle entries (*P* = 0.730), suggesting that the effect wasn’t solely due to decreased behavior during the self-administration session.

**Fig 2:**
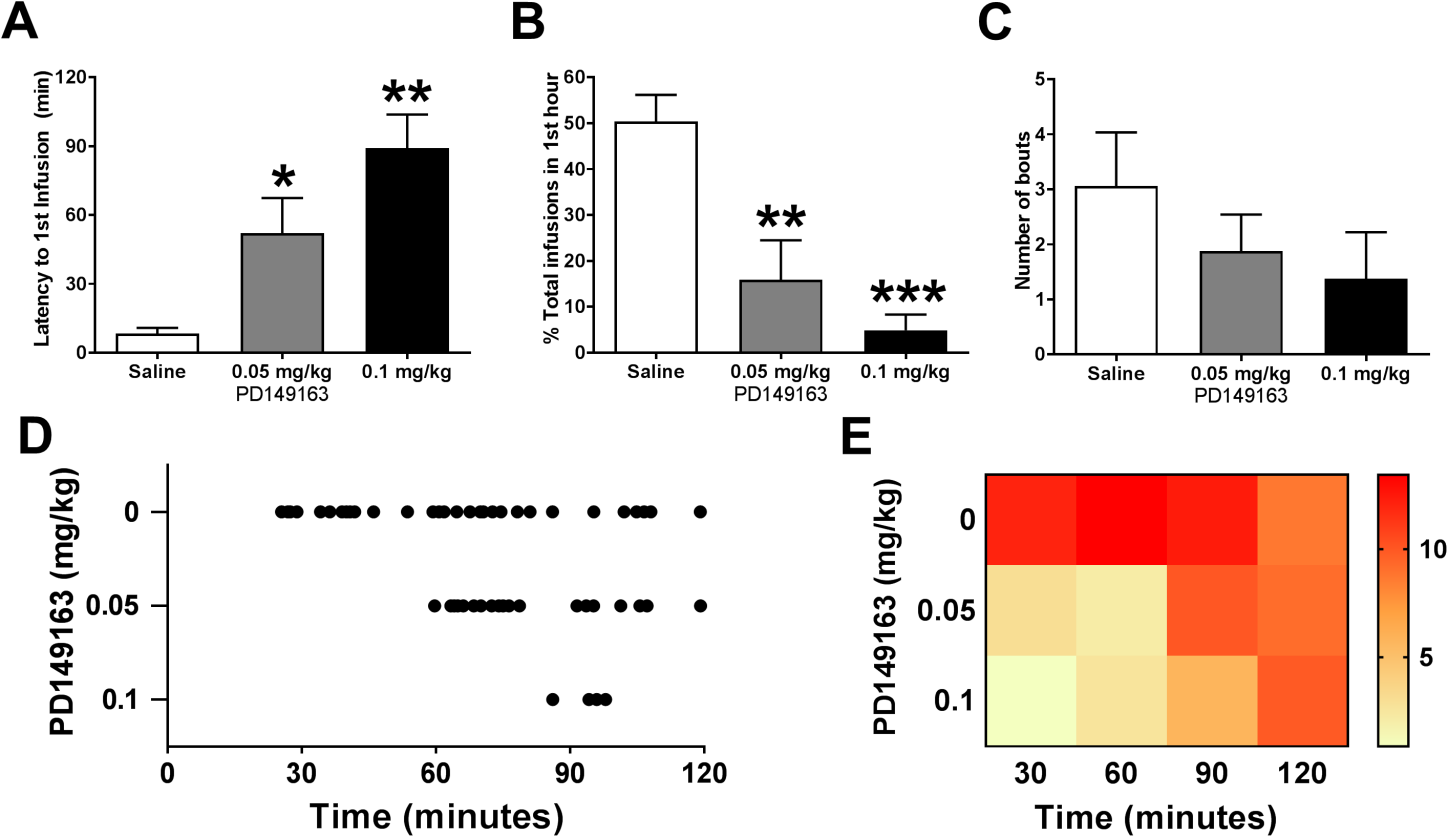
PD149163 alters the pattern of operant responding for METH in mice. An examination of the pattern of responding during the 2-hour operant self-administration session revealed that METH intake was primarily decreased through attenuated behavior early in the session. (A) The latency from the start of the session until the first infusion of METH was significantly increased following PD149163 administration at both doses tested. * *P* < 0.05, ** *P* < 0.001 versus saline, Dunnett’s multiple comparison test. (B) The percent of total infusions earned during the first hour of the session was significantly decreased at both doses of PD149163 tested. ** *P* < 0.001, *** *P* = 0.0001 versus saline, Dunnett’s multiple comparison test. (C) PD149163 did not significantly alter the number of bouts of METH self-administration (defined as 2 or more infusions with less than 120 s between infusions; *P* = 0.055, One-way RM ANOVA), although there was a trend towards a decreased number of bouts. (D) A sample raster plot showing METH infusions (displayed as dots) during the self-administration session for one mouse following saline, 0.05, and 0.1 mg/kg PD149163, showing the delay in responding following PD149163 treatment. (E) A heat map analysis for all mice showing the number of nose pokes in 30 minute bins during the 2-hour self-administration session. Nose poking in the active hole (warmer colors) was delayed with increasing doses of PD149163.

To better characterize the effect of PD149163 on METH self-administration, we examined the pattern of responding during the 2-hour operant session. The latency from the beginning of the session until the first infusion of METH was significantly affected by PD149163 (F_2,14_ = 12.6, *P* = 0.0007), with a significant increase from saline at both doses tested (Fig 2A). Similarly, there was a significant main effect of treatment (F_2,14_ = 21.0, *P* < 0.0001) on the percentage of total number of infusions earned per session that were earned in the first hour of self-administration, with significant decreases at both doses of PD149163 tested (Fig 2B). The number of bouts (defined as at least 2 infusions with ≤ 120 seconds between reinforcers) per session showed a trend towards significance (Fig 2C; F_2,14_ = 3.6, *P* = 0.055), however, the power may have been insufficient to observe a significant change. Representative cumulative records for one mouse (Fig 2D) show how self-administration behavior was both decreased and delayed following PD149163. A heat map analysis of infusions averaged into 30 minute bins for all 8 mice also shows the shift in self-administration following PD149163 treatment compared to saline (Fig 2E).

### Open field locomotion

**Fig 3:**
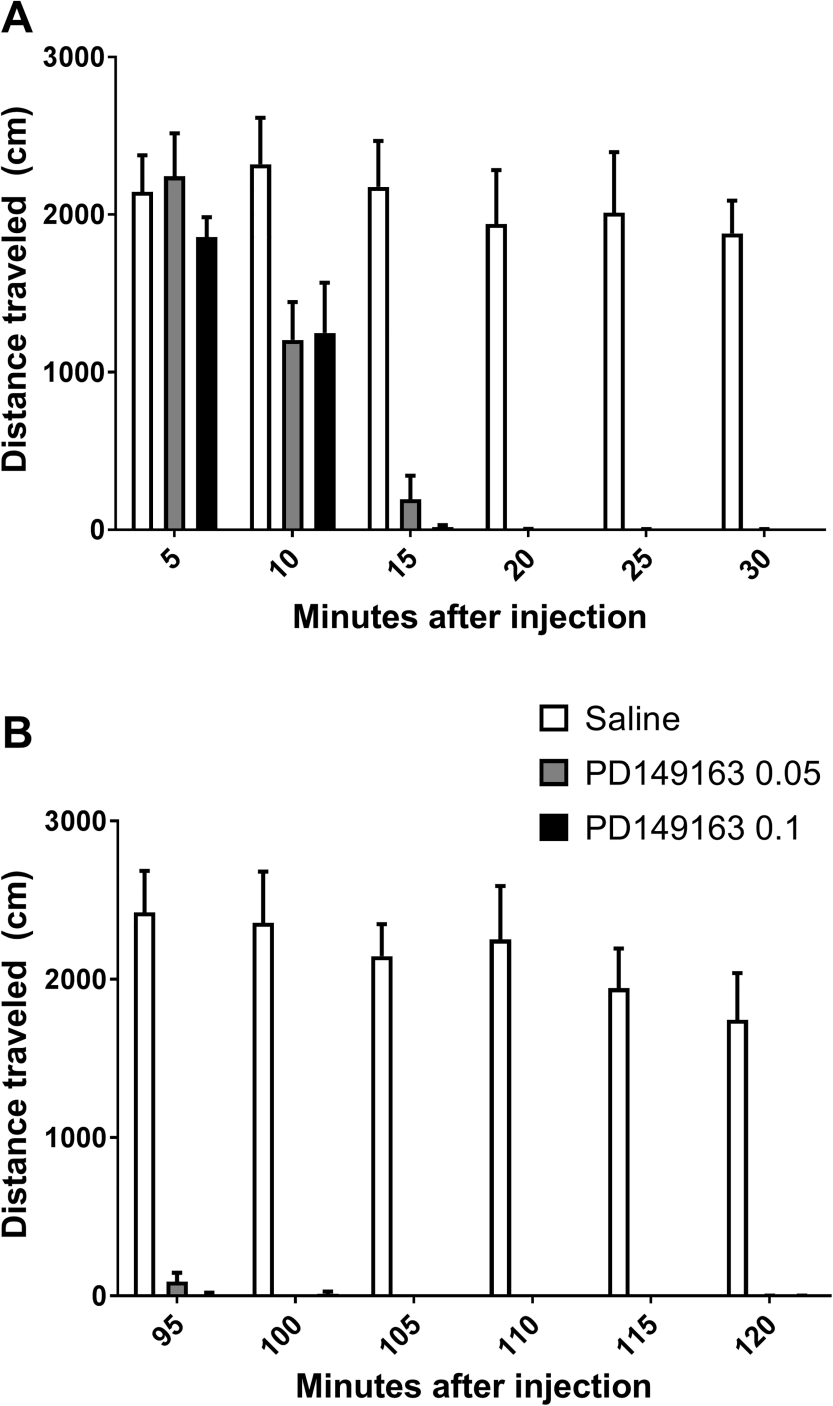
PD149163 impairs voluntary locomotion in an open field beginning 10 minutes until at least 120 minutes following injection. (A) Horizontal distance traveled during the 30-minute session in 5-minute bins beginning immediately following PD149163 injection. (B) PD149163 at both doses tested almost completely eliminated voluntary locomotion 90–120 minutes after injection.

The delay in self-administration behavior combined with experimenter observations of sedation after the operant sessions when PD149163 was administered led us to examine the effect of PD149163 on open field locomotor activity. METH-naïve mice (n = 6) were tested for locomotor activity using a within-subject design, with each mouse tested at two doses of PD149163 and two time points (0–30 minutes after injection and 90–120 minutes after injection). A significant decrease in locomotor activity was seen at both 0–30 minutes (Fig 3A; main effect of time: F_5,20_ = 60.0, *P* < 0.0001; main effect of treatment: F_2,8_ = 28.1, *P* = 0.0002; interaction time x treatment: F_10,40_ = 9.8, *P* < 0.0001) and at 90–120 minutes (Fig 3B; main effect of time: F_5,20_ = 10.4, *P* < 0.0001; main effect of treatment: F_2,8_ = 64.4, *P* < 0.0001; interaction time x treatment: F_10,40_ = 8.4, *P* < 0.0001).

### Rotarod

**Fig 4:**
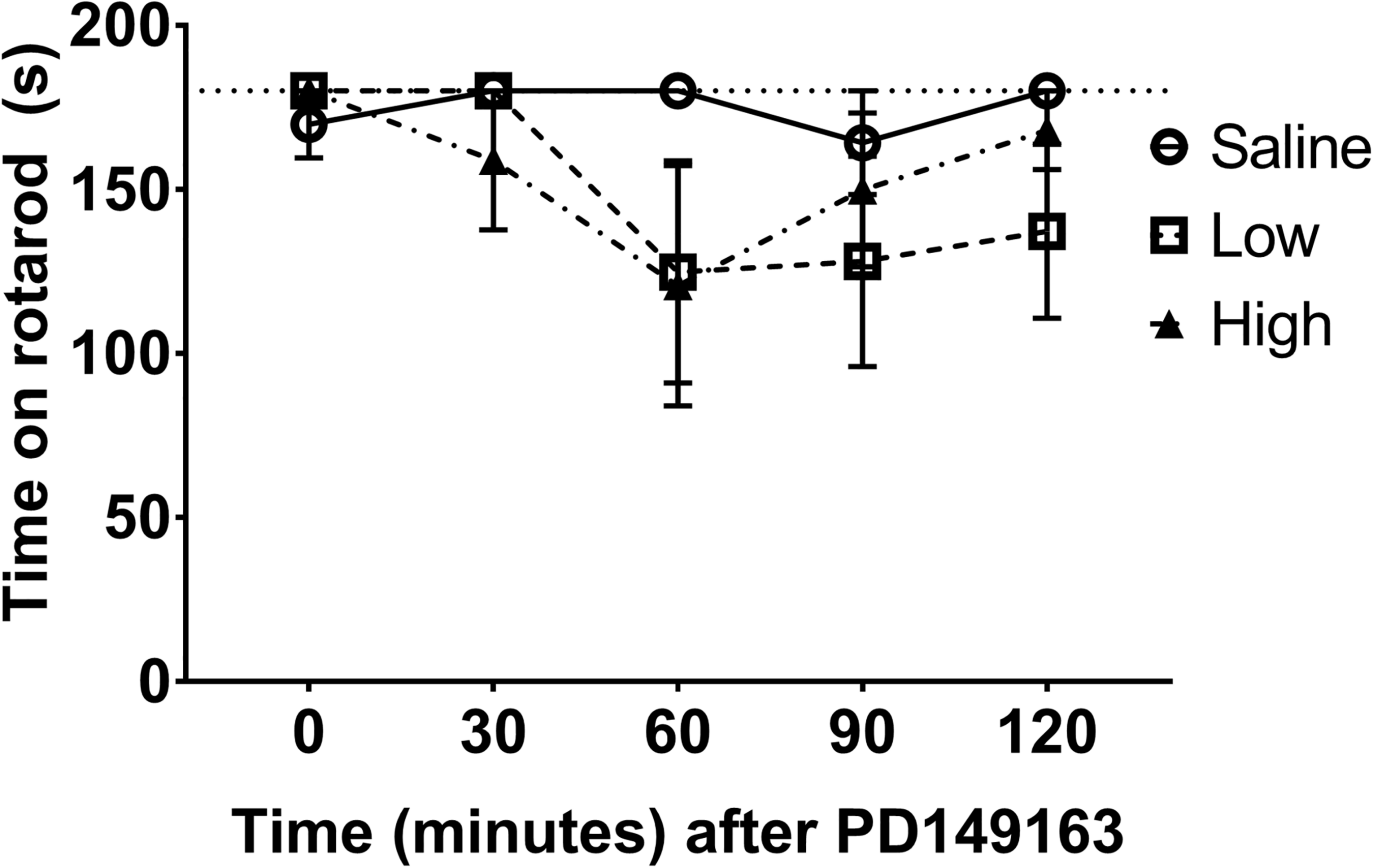
Rotarod performance is non-significantly impaired by pretreatment with PD149163. Mice injected with PD149163 (0, 0.05, 0.1 mg/kg) showed no significant main effect of drug (*P* = 0.08) or time (*P* = 0.38) on their ability to stay on a fixed speed rotating rod for up to 2 hours after drug injection.

While we observed substantial deficits in open field locomotor activity, previous studies have reported no [6] or little [11] effect of systemic PD149163 (0.1 mg/kg) on rotarod performance. In a within-subjects design, adult, male METH-naïve mice (n = 5) were treated with 0, 0.05, and 0.1 mg/kg PD149163 and tested for performance on a fixed speed rotarod at 30 minute intervals for 2 hours after injection. Although there was a slight decrease (~30%) in time spent on the rotarod that peaked at 60 minutes post-injection (Fig 4), there was not a significant main effect of time (F_4,16_ = 2.54, *P* = 0.08) or dose (F_2,8_ = 1.10, *P* = 0.38).

## Discussion

Neurotensin is an endogenous peptide that is present in both the gut and brain. It is implicated in reward and modulation of neurotensin has been touted as a putative treatment for drug addiction (for review see [24–25]). While previous studies have reported a decrease in psychostimulant self-administration following systemic injection of neurotensin or neurotensin receptor agonists [6, 7], there was little insight into how neurotensin receptor agonists decrease the pattern of self-administration or if these results could be replicated in non-food deprived animals. Results from the present study suggest that systemic treatment with the NTS1 receptor agonist PD149163 decreases METH self-administration in ad libitum fed mice during daily limited access sessions. The self-administration of METH intake after PD149163 injection is similar to previously published results reporting decreased self-administration of food [16–19], nicotine [20], and METH [6–7]. The current study extends previous published effects of neurotensin receptor agonism on METH intake by looking at non food-deprived animals and the pattern of self-administration to probe whether PD149163 decreases responding during initiation or maintenance of self-administration in mice with stable self-administration behavior.

While the session-long decrease in self-administration is significant at both doses of PD149163 tested, the pattern of self-administration suggests a trend towards a dose-dependent response with the higher dose resulting in a longer latency until first infusion earned (*P* = 0.089, low vs. high dose PD149163; see Fig 2). The almost complete lack of initiation of self-administration in the first hour following PD149163 suggests that the drug does not block or amplify the subjective effects of METH (as the subjects didn’t actually self-administer any METH until hour two), but rather is drastically decreasing motivation to gain access to METH. One possible explanation is that the mice have increased satiety that causes a decrease in self-administration initially in the self-administration session. This is consistent with neurotensin’s ability to reduce food intake in ad libitum fed mice early in the dark portion of the light cycle, suggesting enhanced satiety [16]. Doses of neurotensin capable of decreasing food intake do not, however, condition a flavor aversion [19]. We observed that METH self-administration resumes as PD149163 begins to wear off after 60–90 minutes, similar to anorectic effects observed previously [16–17].

Similar to our open-field locomotor activity results, previously published work reported a decrease in voluntary, open-field locomotor activity in rodents following administration of neurotensin or neurotensin receptor agonists [11, 26]. The inability to locomote would be a sincere confound to self-administration. Thus, to rule out frank inability to move during the initial portion of the self-administration session after systemic administration of PD149163, we examined motivated locomotion using the fixed speed rotarod. Our findings were in line with other published reports of no [6] or a small (0.1 mg/kg PD149163, [11]) decrease in locomotor ability when measured on a rotarod. The rotarod data strongly suggest that the mice were capable of movement and METH self-administration during the operant sessions. In support of our rotarod data, operant responding for METH (Fig 2D and 2E) returned during the second hour to rates similar to saline control, suggesting that the mice were indeed capable of motivated behavior. In addition, although mice were generally very calm and not stimulated at the end of the session (locomotor and self-administration), they were awake and able to move when placed in the operant chamber 30 minutes after injection (observation, data not shown). Previous studies have shown that PD149163 does not induce catalepsy, even at much higher doses than tested here [27]. It is possible that an impairment in locomotive ability contributed to the decreased self-administration, but we believe that motivation to self-administer METH was a major contributor to the decreased intake.
